# 14 Prognostic Factors for In-hospital Mortality of Geriatric Burns from the US National Inpatient Sample 2016-2018

**DOI:** 10.1093/jbcr/irac012.018

**Published:** 2022-03-23

**Authors:** Yangtian Yi, Sebastian Q Vrouwe, Lawrence J Gottlieb, Daniel S Rubin

**Affiliations:** The University of Chicago Pritzker School of Medicine, Chicago, Illinois; University of Chicago, Chicago, Illinois; University of Chicago, Chicago, Illinois; University of Chicago, Chicago, Illinois

## Abstract

**Introduction:**

Older adults experience a disproportionately higher rate of hospitalization and mortality due to burns. However, national estimates of in-hospital mortality in geriatric patients with acute burns outside of the recognized burn centers are lacking. The purpose of this study is to characterize such national estimates and identify the prognostic factors associated with in-hospital mortality in elderly patients with acute burn injuries.

**Methods:**

Patients ≥50 years with an acute burn diagnosis in the United States National Inpatient Sample from 2016 to 2018 were identified by using International Classification of Diseases, Tenth Revision, Clinical Modification codes (T20.0-T31; initial encounters only). Trend weights were used from the Agency for Healthcare Research and Quality to generate national estimates. Factors associated with in-hospital mortality were evaluated by multivariable logistic regression.

**Results:**

A total of 60,515 weighted discharges met inclusion criteria. For age groups 50-64 (reference category), 65-74, 75-84, and ≥85, the numbers of discharges were 33,100, 15,295, 7,880, and 4,240, respectively; the in-hospital mortality rates were 3.3%, 5.3%, 6.6%, and 9.9%, respectively. The full list of significant variables of the multivariable regression can be seen in Table 1. Specifically, variables associated with increased in-hospital mortality include age 75-84 (odds ratio [OR], 3.91; *p*< 0.001), age ≥85 years (OR, 11.94; *p*< 0.001), total body surface area (%TBSA) 10-19% (OR, 2.93; *p*=0.017), and %TBSA ≥20% (OR, 27.78; *p*< 0.001). Intentional burns were associated with an increased risk of mortality (OR, 7.86; *p*=0.005), whereas hot liquids and vapor burns were associated with a decreased risk of mortality (OR, 0.33; *p*=0.043). Respiratory distress/failure (OR, 5.78; *p*< 0.001), aspiration pneumonia/pneumonitis (OR, 5.01; *p*=0.004), shock (OR, 4.58; *p*=0.004), and acute renal failure (OR, 2.85; *p*=0.005) were associated with an increased risk of mortality. One or more surgical excisions were associated with a reduced risk of mortality (OR, 0.26; *p*< 0.001).

**Conclusions:**

Age ≥75 years, %TBSA ≥10%, intentional burn as the mechanism of injury, and certain comorbidities and complications were significant risk factors for in-hospital mortality in geriatric burn patients.

